# Reddit as a Social Media Self-Management Tool for Inflammatory Bowel Disease: Qualitative Analysis

**DOI:** 10.2196/75137

**Published:** 2025-08-01

**Authors:** Tosin Adeyemi, Brian Gutermuth, Gal Hodish, Jeffrey Berinstein, Kira Newman, Sarah Hawley, Minal Patel, Peter Higgins, Ken Resnicow, Sarah Krein, Shirley Cohen-Mekelburg

**Affiliations:** 1 University of Michigan Medical School, Ann Arbor, MI, United States; 2 Department of Internal Medicine, Michigan Medicine, University of Michigan, Ann Arbor, MI, United States; 3 Division of Gastroenterology, Michigan Medicine, University of Michigan, 1500 East Medical Center Drive, Ann Arbor, MI, 48109, United States, 1 734 962 5000; 4 VA Center for Clinical Management Research, VA Ann Arbor Healthcare System, Ann Arbor, MI, United States; 5 Division of Health Education and Health Behavior, University of Michigan School of Public Health, Ann Arbor, MI, United States

**Keywords:** self-management, social media, community, Crohn disease, ulcerative colitis, quality of life

## Abstract

**Background:**

Given the widespread discussion of inflammatory bowel disease (IBD)–related topics on social media, these platforms play an important role in increasing our understanding of the needs, preferences, and experiences of patients living with IBD.

**Objective:**

This study aims to develop an in-depth understanding of Reddit (Reddit, Inc) social media discussions related to individuals’ experiences and perspectives living with and managing inflammatory bowel disease.

**Methods:**

We identified threads on the r/IBD, r/Ulcerative Colitis, and r/CrohnsDisease subreddits between April 2022 and 2024 using *a priori* selected keywords to identify IBD-related experiences, perceptions, and needs. We used rapid qualitative analysis using preselected keywords (deductive) followed by further domain development (inductive) to generate a broad understanding of Reddit discussions related to patients living with IBD.

**Results:**

We identified 659 posts comprising 207 (31.4%) original and 452 (68.6%) reply posts related to patients living with IBD. Findings highlighted that people use Reddit to seek knowledge, advice, empathy, and validation as well as to share experiences in five key areas related to: (1) symptoms as a major burden of patients living with IBD; (2) experiences with medications and concerns about treatment choices; (3) health care–related challenges; (4) dietary regimens and dietary guidance for handling symptoms and overall treatment; and (5) negative impact of IBD on mental health.

**Conclusions:**

Use of social media as an avenue for patients with IBD to gain knowledge, empathy, and support suggests that traditional clinical care does not fully address the needs of patients living with IBD as it relates to IBD knowledge, advice, empathy, validation, and sharing of experiences. While social media platforms can provide a platform for patients to share their experiences and build community, study findings also inform future efforts to address identified gaps in care, including the development of interventions to assist patients in managing their symptoms, diet, medications, health care, and mental health concerns.

## Introduction

Inflammatory bowel disease (IBD), including its subtypes of Crohn disease and ulcerative colitis, is a chronic inflammatory condition of the gastrointestinal tract that affects over 3 million Americans and 4.9 million people worldwide [1]. Patients with IBD experience gastrointestinal symptoms, such as abdominal pain, diarrhea, and bloody stools, as well as extraintestinal symptoms such as fatigue, arthralgias, rashes, and mood disturbances that impact their daily lives [2]. Self-management in IBD refers to the daily work required by patients to keep their disease under control and minimize its impact on their physical and psychological health [3]. Effective self-management helps patients and their clinicians recognize early changes in symptoms and optimize medical therapy to improve health outcomes and reduce health care overuse. Systematic review data suggest that effective self-management in IBD supports IBD-specific knowledge, enhances social participation, improves health-related quality of life, and reduces health care usage [4,5]. Nonetheless, determining how best to provide patients with the support and resources that they need for daily IBD care and effective self-management requires a better understanding of the experiences, preferences, and needs of patients living with IBD.

Many patients with IBD (as with other conditions) rely on the internet and social media platforms (eg, X [formerly Twitter], Facebook [Meta], Reddit) for health information, advice on self-management, sharing of personal experiences, and social support [6,7]. Engagement across social media has grown significantly, with 86% of internet users reporting some social media activity, including sharing health information, participation in online support groups, or viewing health-related videos [6]. In fact, patients with IBD report that they are spending 30‐60 minutes each day on social media websites [7]. Patients with IBD are also increasingly engaging with conversational large language models, such as ChatGPT, to seek knowledge and advice on specific clinical questions and concerns. In one study, ChatGPT demonstrated some scientific reliability in answering the top 10 most common questions from a group of gastroenterologists, ranging from curability of the IBD to transmission to children [8]. However, some outputs were incorrect. The risk of incorrect responses (ie, artificial hallucinations) from these large language models limits their reliability for patients, and their potential role in providing empathy or shared experience is unclear. While the reliability of information communicated on social media also varies, social media interactions may play a more likely role in addressing patients’ needs for sharing of experiences [9].

Several recently published studies have increased our understanding of the role of social media platforms in the experiences of patients with IBD. One study identified symptoms, medications, and nutrition as the most common distress-related IBD topics discussed on Reddit and X (formerly Twitter, X Corp) [10]. A second study explored patients’ experience with IBD flares by examining flare-related posts on several online ulcerative colitis forums. They found that these online forum posts most frequently discussed treatment experiences, treatment side effects, and symptoms [11]. Another study using social media data described the experiences and knowledge of the risks and benefits of IBD-related biologic treatment and individual medication decision-making [12].

Given the widespread discussion of IBD-related topics on social media, these platforms play an important role in increasing our understanding of the needs, preferences, and experiences of patients living with IBD. Therefore, we aimed to develop a more in-depth understanding of Reddit social media platform discussions related to individuals’ experiences, perspectives, and preferences in living with and managing IBD. Study findings will inform the development of tailored self-management tools that support patients living with IBD to improve health outcomes.

## Methods

### Study Design and Population

**Figure 1.**
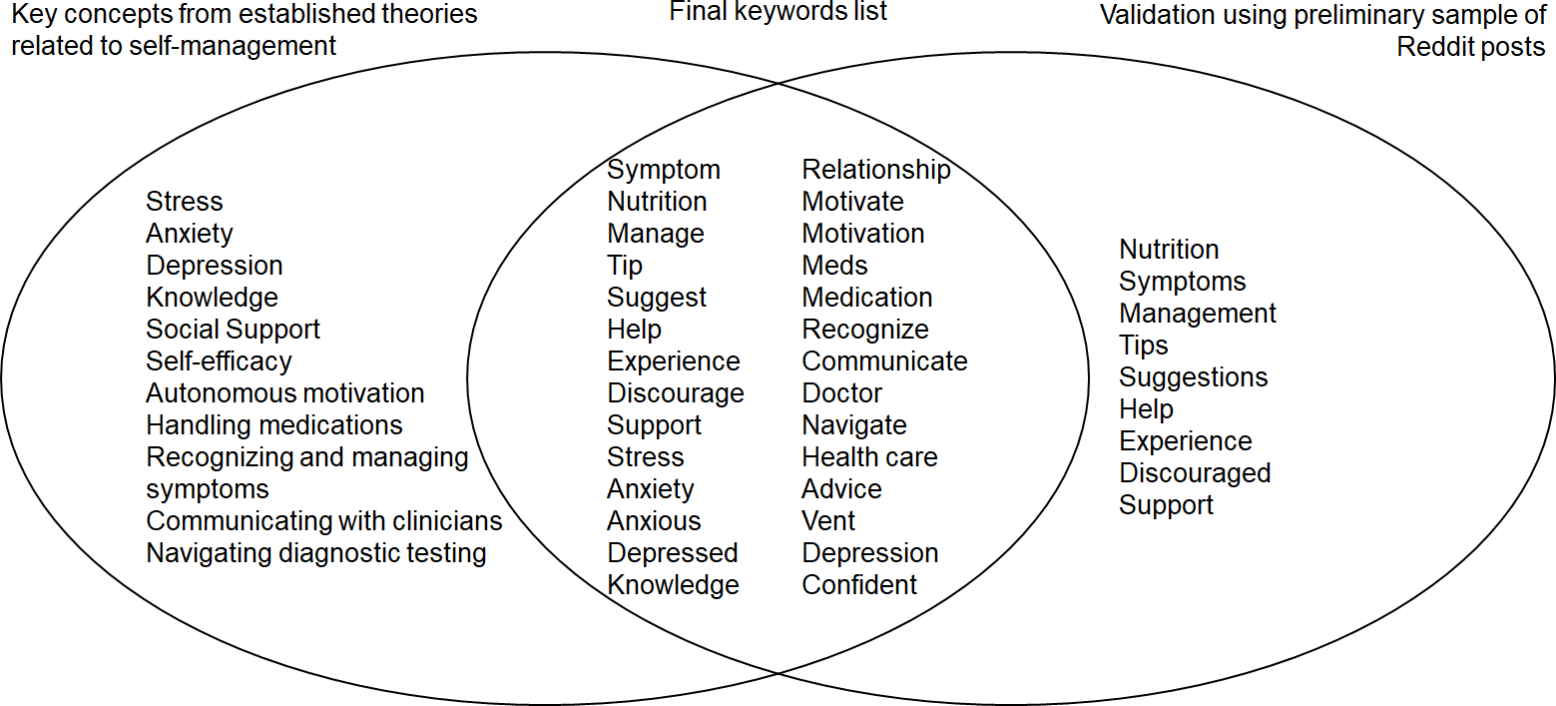
A priori selected keywords to identify discussions about experiences, perceptions, or needs related to living with inflammatory bowel disease.

Reddit is a web-based social media platform founded in 2005 with over 70 million daily active users [13]. People can anonymously post on the platform and form communities surrounding a specific topic of interest [13]. Topics are organized into communities known as *subreddits*, which are comprised of individual discussions known as *threads*. For the topic of IBD, the most popular subreddits include “r/IBD” (25,000 members), “r/UlcerativeColitis” (43,000 members), and “r/CrohnsDisease” (67,000 members). Data collection was conducted between April and June 2024. We identified original threads on the r/IBD, r/Ulcerative Colitis, and r/CrohnsDisease subreddits between April 2022 and April 2024 using Brandwatch (Cision Group Ltd), a commercially available social media management tool [14]. We included posts with *a priori* selected keywords to identify discussions about experiences, perceptions, or needs related to patients living with IBD. Keywords included symptom, nutrition, manage, tip, suggest, help, experience, discourage, support, stress, anxiety, anxious, depression, depressed, knowledge, relationship, motivate, motivation, medications, medication, recognize, communicate, doctor, navigate, health care, advice, vent, and confident (Figure 1). These keywords were selected from published data on self-management principles, including Lorig self-management model [3], social cognitive theory [15], and self-determination theory [16], and finalized by reviewing a preliminary sample of IBD subreddit posts for keywords. Original posts and their top 3 reply posts were included. Posts were excluded if related to IBD diagnostics, irritable bowel syndrome, noninflammatory bowel disease (eg, microscopic and lymphocytic colitis), or deemed not relevant (eg, “thank you!” reply post).

### Qualitative Analysis

We used a rapid qualitative analysis approach to generate a broad understanding of Reddit social media discussions and experiences related to patients living with IBD. Rapid qualitative analysis is a method for efficiently analyzing qualitative data in a systematic manner to gain a preliminary understanding of a large amount of data [17]. Study design, data collection, and analysis were informed by a consensus-based framework, that is, Planning for and Assessing Rigor in Rapid Qualitative Analysis (PARRQA), (Table S1 in Multimedia Appendix 1) [18]. Members of the study team first developed a summary template using Microsoft Excel that focused on several key domains derived from our conceptualization of IBD self-management and key constructs from Lorig self-management model, social cognitive theory, and self-determination theory, including medical management, role management, emotional management, motivation, autonomy, and self-efficacy [17,19]. ^-^The final domains tailored to IBD included symptom management, medication management, navigating healthcare, nutrition, social relationships, and emotional health. Posts could fall under multiple domains, and we also included an open domain for relevant data that did not align with one of the key domains. Two members of the study team (TA and SCM) then used the template to create summaries of each post, with an initial set of posts and summaries (96 original posts and 61 replies) reviewed by both team members to establish consistency in data interpretation during a training and calibration period. The remaining posts were then summarized independently. The summaries were reviewed and discussed at team meetings to identify patterns within and across domains and to address any discrepancies. The agreement was derived through consensus among team members, and any discrepancies were resolved via discussion.

### Ethical Considerations

This study leveraged publicly available deidentified data, and as such, informed consent was not required. Further, this study was deemed exempt by the University of Michigan Institutional Review Board (IRB number HUM00244957).

## Results

### Overview

We identified a total of 659 posts comprising 207 (31.4%) original posts and 452 (68.6%) reply posts on the r/IBD, r/UlcerativeColitis, and r/CrohnsDisease subreddits between April 2022 and April 2024 related to patients living with IBD that were included in the analysis. Findings highlight that people use Reddit to seek knowledge, advice, empathy, and validation as well as to share experiences related to several daily challenges. In alignment with the summary template, these challenges were focused on five key domains: (1) symptoms as a major burden of patients living with IBD; (2) experiences with medications and concerns about treatment choices; (3) health care–related challenges; (4) dietary regimens and dietary guidance for handling symptoms and overall treatment; and (5) the negative impact of IBD on mental health (Table 1).

**Table 1.** Reddit is used to seek knowledge, advice, empathy, validation, and to share experiences and challenges related to living with inflammatory bowel disease in five key domains.

Domain	Representative quotes
Domain 1: Symptoms are a major burden of living with IBD.^a^	
1.1 Sharing experiences with symptoms	“Since today morning, I’ve had almost 5 bloody diarrhea stools, the highest I’ve ever encountered. Taking steroids (…2 mo’s) and also Imuran…last 2 days…what next pls help.” “I had a maintenance colonoscopy a few days ago, and like every time before, I’m back in a flare up. Most of the time I just grin and bear it, cry silently where no one can see, sort of thing.” “…I have an 8 month old daughter who I feel like I’m failing by not having the energy I should have for her. My husband is so patient, and I feel like he deserves better. A normal spouse who is happy and healthy…” “…My boss informed me that I am now on probation, as I have called out 19 times since February…This is just not feasible for me. Never mind my colitis not being in remission...”
1.2. Seeking advice with handling symptoms	“…fewer trips to the bathroom daily…but have really struggled with fatigue…I’m wondering…if anyone has advice...I’m also seeing a specialist…but I was just curious about anyone’s personal experiences.” “I experience this thing where my stomach will cramp up horribly and then I’ll get the runs...I also feel extremely tired afterwards. Not sure what to do to make this stop but when I brought it up to my GI she didn’t say much…Who else experiences this and what do you do for relief?”
1.3. Seeking validation and empathy for symptoms	“Hi, I’m on Prednisone…I’ve been getting some hand tremors recently and not sure if it’s related. The shaking today is unbearable, and it comes with a bad jittery feeling. Tell me I’m not crazy!” “…Crohn’s can be so gross and so off putting…having a place like Reddit or another chronically ill friend can be helpful to have the “dark thoughts” without feeling judgment can be really liberating.”
Domain 2: Experiences with medications and concerns about treatment choices	
2.1. Sharing negative experiences with treatment and concerns about treatment failure	“…I quit. I’ve been in a flare for almost a year…The prednisone isn’t working and it’s destroying me. I’m not sleeping, I’m the most uncomfortable I’ve ever been with inflammation and bloating, I’m having some scary thoughts that are frightening me…I’ve literally done everything I can think of but this has overtaken my life and ruined it.” “…Humira was ditched because my ocd got good kicks from it.” “If this medicine fails, I’ve failed them all…and will probably be recommended to have surgery, which I’ll refuse to get until the day I’m developing cancer or something.” “…I got my Stelara loading dose 6 weeks ago and have barely noticed a change. It’s sometimes just a little better, but then it fluctuates back to its worst...How long does Stelara take? Is this a sign I’m failing?”
2.2. Seeking advice about medication choices	“I was diagnosed with ulcerative colitis endoscopic biopsy said it was non-specific colitis. The doctor asked me to take mesalazine 500mg for a month only, three tablets daily. What do you think…?”
2.3. Sharing positive treatment experiences to provide reassurance	“Many of us have fallen into this trap. Stopped bleeding and mucus is gone. I’m good to go. No more meds I’m cured! Nope, everything just comes back with a vengeance!” “…You’ve got this & once you get over the actual surgery, I’m sure that your quality of life will be massively improved. Don’t be afraid to ask your team as many questions as you need to feel satisfied & comfortable. And there’s no shame in asking for help...Good luck &, if you feel up to it, keep us updated.”
2.4. Sharing treatment strategies and curiosity about non-pharmacologic options	“…Basically, all meds including pred don’t work for me except this...So for this recipe you will need…either Mesalazine enema, warm water, generic up bum enema saline solution…I tend to do it for a few days then stop and repeat when/if symptoms return…”
Domain 3: Healthcare-related challenges	
3.1. Sharing frustrations and difficulties accessing their gastroenterology team	“…I have work tomorrow and I need to call in sick, then call the GP and then call my specialist IBD team. THREE different people who I need to repeat myself to. It’s just so draining.” “…I’ve been going back and forth the last month between feeling kinda ok and feeling like I’m getting way worse…I have a doctor’s appointment in 5 days, but I feel like…I’m just sitting around not doing anything about it...” “Before anyone says, ‘did you ask your doctor… if she was accessible and helpful I wouldn’t be on reddit...”
3.2. Seeking advice about affordability of medications	“It all seems expensive as hell…I do not make a lot of money at my job. This is huge for me. Is there any way to get it cheaper? I am newly diagnosed and just feeling overwhelmed.”
3.3. Sharing frustrations with not feeling heard by the medical team	[after trialing multiple rounds of prednisone] “scream and cry to my doctors for help beg for testing…told it was in my head and put on some antidepressant that gave me heart palpitations.” “Solidarity…My weight has been a struggle since my surgery...Thankfully the PA I see at my gastro’s office believes me and doesn’t just see the weight like so many others before. It’s nice to have one medical professional after all these years who understands that I can’t eat vegetables…I can’t just go on walks.”
3.4. Sharing hesitation with seeking advice from the medical team	“…Just wondering if I should do one of these saline enemas or go to the doctor. Really don’t wanna visit the doctor tho.” “I am not a lab rat.”
Domain 4: Dietary regimens and dietary guidance for handing symptoms and overall treatment.	
4.1. Sharing experiences with diet	“…I’m so tired of carefully picking everything I eat, every ingredient… I basically live on meat, dairy and supplements. I want to believe I could eat more foods one day without getting sick but I’m worried….”
4.2. Seeking advice on diet	“I still have a lot of “healthy” foods i can’t tolerate despite my Crohn’s being relatively behaved…when i see dietitians it’s a JOKE because they give me either all the foods i can’t eat… I’m just curious if anyone else has had similar circumstances and has advice for me…” “With medication and a disciplined diet, you can end up living like a well-oiled machine and calculated risks will be your choice.”
Domain 5: IBD’s negative impact on mental health	
5.1. Seeking advice on comorbid anxiety and depressed mood	“…I’m a very stressed out anxious person and i feel like it just makes my Crohn’s 100x worse. I worry when i feel unwell, i worry when i feel well that I’m going to get unwell, it stops me from functioning…” “I’ve noticed that my anxiety ALWAYS makes me need to use the restroom...I’m wondering if anyone has tried anxiety medication and seen any positive results?”
5.2. Sharing feelings of defeat and embarrassment	“…I just don’t have it in me to put up the front all day. I hate this bullshit incognito fucking disease. I’m so tired of feeling tired. I didn’t plan on sharing this much but this was more therapeutic than I expected… “…It feels like another way my youth is being stolen by this disease…Does anyone else find themselves frequently thinking about how UC is prematurely aging them?” “I am so tired of not being taken seriously because no one can ‘see’ my Crohn’s…” “Stay positive and remember that your (A) not alone, (B) there is meds to help and (C) believe mentally that you can overcome this battle, and you shall.”

a IBD: inflammatory bowel disease

### Domain 1: Symptoms Are a Major Burden of Patients Living With IBD (28% of Posts)

#### Sharing Experiences With Symptoms

Individuals described concerns about a variety of IBD-related gastrointestinal symptoms, including bloody stools, diarrhea, constipation, urgency, nausea, vomiting, and bloating. Posts frequently reported abdominal pain or pain from abscesses. One individual described their abdominal pain as, “I can feel my insides throbbing if that makes sense.” In contrast, other posts reported on minimal pain and feeling healthy. This discussion of symptoms seemed to especially revolve around medication use, particularly steroid tapers. One post described the first day after tapering off prednisone as “unbearable,” with significant abdominal pain, urgency, and bloody stools. Extraintestinal symptoms, especially fatigue, were also commonly discussed. Fatigue and hair loss were commonly reported as symptoms of a flare, though some posts also blamed medications for these effects. For example, one individual with Crohn disease described fatigue as a side effect of azathioprine. Overall, individuals’ symptom burden hindered their ability to spend quality time with others. For example, one post described not being able to play on the floor with their son because of a nonhealing abscess. Another post described difficulty conversing with friends while experiencing flare symptoms. People also shared experiences of gastrointestinal symptoms hindering intimacy with their partner. Posts discussed the challenges of working with gastrointestinal symptoms. For example, one person noted that it was distracting for coworkers to see them in pain or make frequent bathroom trips. Others described having to stop working due to lack of work accommodations. In response, reply posts normalized these experiences and advised honesty in communicating symptoms with others.

#### Seeking Advice With Handling Symptoms

Many individuals sought others’ experiences to understand whether their own symptoms are manifestations of an IBD flare, medications, or another etiology. For example, several posts described worsening gastrointestinal symptoms following new medication initiation and were seeking opinions on the underlying etiology. One post described developing bloody stools after discontinuing marijuana and inquired whether this was related. People also commonly sought others’ experiences with symptom treatment. One post described improved urgency with mesalamine and prednisone but inquired about ways to address residual fatigue. The user specifically noted that they were planning to ask their gastroenterologist but wanted to also hear personal experiences. In turn, several posts provided pain treatment recommendations, including guided meditation, deep breathing, cannabidiol oil, heating pads, or antispasmodic medication for cramping. Other posts provided nausea treatment recommendations, such as flavorful foods, essential oils, hot showers, or antiemetic medication. Posts also promoted self-monitoring of symptoms, allowing for trending symptom changes. However, one post emphasized that self-monitoring of symptoms was not sufficient and encouraged clinician input.

#### Seeking Validation and Empathy for Symptoms

Many users sought validation for concerns about symptoms and for taking time off work due to symptoms. Even individuals who minimized their symptoms and described their IBD experience as less severe than others also emphasized that they still found it difficult to control. Often, reply posts provided encouragement and validation for these symptoms and experiences.

### Domain 2: Experiences With Medications and Concerns About Treatment Choices (46% of Posts)

#### Sharing Negative Experiences With Treatment and Concerns About Treatment Failure

Posts described trialing multiple IBD-targeted medications in pursuit of better disease control but experiencing adverse effects, such as fatigue, night sweats, and cramping. These adverse effects were particularly frequent among prednisone users. Individuals also expressed worry about treatment failure. Treatment failures led to disappointment and worsened mental health. One post described an initial response to a new medication, but a subsequent return of symptoms. The idea of initiating new medications brought uncertainty and anxiety. This concern was often directed towards surgery avoidance. In contrast, others were frustrated by their symptoms and felt that they were at the point of asking for surgery.

#### Seeking Advice About Medication Choices

Individuals sought others’ experiences with medications, including onset of action, adverse effects, and treatment response. In response, posts expressed empathy and encouragement related to medication. For example, one post described an individual’s experience with medication-refractory ulcerative colitis, acknowledged their frustration, and expressed hope for an effective medication treatment in the future. Other users sought advice or provided practical aid with medication administration, including how to taper prednisone and store medicines, and when to stop a medication. Finally, people also sought assurances on specific medications recommended by clinicians.

#### Sharing Positive Treatment Experiences to Provide Reassurance

While some posts described negative experiences with treatment, others posted about their positive experiences with medications leading to long-term disease control. One post described that while it took time, initiation of a biologic “gave them their life back.” A second post admitted initial hesitancy to start biologic treatment, but the person ultimately decided it was the “best course” after researching risks and benefits. Similar posts provided reassurance that patients should be prepared for trial and error to find an effective medication. Other posts commented on the convenience of self-injection over infusions and the importance of maintaining treatment to prevent recurrent flares. One post described an individual’s experience with a bowel resection and encouraged others that surgery can provide a good quality of life when medications are ineffective.

#### Sharing Treatment Strategies and Curiosity About Nonpharmacologic Options

Several posts inquired about nonmedication treatment options, often related to preferences for a “natural” option or avoiding adverse effects. One post described wanting to avoid medication because of “all the bad things I have heard about it.” Other users sought adjuncts to medication such as herbal treatment or physical therapy. Some posts also describe alternative treatments (eg, homemade enemas) while awaiting medication approvals.

### Domain 3: Health Care–Related Challenges (21% of Posts)

#### Sharing Frustrations and Difficulties Accessing Their Gastroenterology Team

Several posts described frustration with navigating health care and, in particular, with delays in accessing their medical team and needing to coordinate multiple clinicians. Perceived delays in care are common. Posts described concerns about symptoms while waiting to follow up with a gastroenterologist posthospital discharge. For example, one post sought advice for ongoing bloody stools with tapering prednisone while waiting to speak with their doctor. Similarly, other individuals described the frustration with being unable to address their symptoms while awaiting a specific medical appointment date or a response from their physician. For example, some people specifically mentioned that they would seek advice from their medical team rather than social media if they were accessible. Several individuals described their experiences with fragmented care. For example, one post described the frustration of their primary care physician directing them to their IBD team, or their IBD team directing them to a rheumatologist. These posts also described feeling “drained” with repeatedly relaying concerns to multiple clinicians. Meanwhile, other posts described intentionally transitioning medical teams as a result of feeling that specific concerns were not addressed or if their regular gastroenterologist was not available. In response, many reply posts encouraged others to find physicians that were up to date on research and could address their concerns.

#### Seeking Advice About Affordability of Medications

Many individuals perceived that insurance companies largely dictated their care. Some specific examples include forced switching of biologics to biosimilars, discontinuing coverage of effective medications, and missing medication doses due to insurance changes. Posts highlighted how insurance-driven medication challenges have led to consequences, such as worsening symptoms. In response, other people shared personal experiences and tips for navigating insurance coverage or opportunities for acquiring less expensive medications.

#### Sharing Frustrations With Not Feeling Heard by the Medical Team

Posts described individuals’ experiences with clinicians dismissing or not fully addressing their symptoms. One individual described an experience with chronic nausea and vomiting that had never been addressed despite its negative impact on their quality of life. Another person sought advice on whether to reach out to their medical team again, and their medical team dismissed their experience transitioning to a liquid diet for symptoms concerning for bowel obstruction. In contrast, other posts described positive experiences with health care, including trust in the medical team and gratitude for clinicians’ understanding of their symptoms and hardships.

#### Sharing Hesitation With Seeking Advice From the Medical Team

Some individuals sought advice on symptom management, but specifically noted a preference for avoiding communication with their medical team. Several reasons were identified for avoiding medical team communication, including fear about next steps, frustration with multiple visits, and anticipated discomfort with lab work or intravenous medications. In reply, posts encouraged individuals to discuss these concerns with their medical team, advised emergency care, and identified specific evaluation tests such as inflammatory markers or colonoscopy.

### Domain 4: Dietary Regimens and Dietary Guidance for Handling Symptoms and Overall Treatment (15% of Posts)

#### Sharing Experiences With Diet

Dietary planning predominated IBD discussions, given the impact of food on symptoms. Individuals described benefits and frustrations with various dietary restrictions aimed at managing symptoms or treating their IBD, including but not limited to gluten-free, dairy-free, high-protein, low-fiber, and elimination diets. Some individuals incorporated vitamins, supplements, and probiotics into their diet. For some individuals, dietary changes alleviated symptoms, while for others, certain foods triggered their symptoms. Specific posts shared experiences with food journaling to track symptoms in relation to diet. Malnutrition was also shared as a common concern. One post described meal prepping to encourage themselves to eat.

#### Seeking Advice on Diet

Given the impact of diet on symptoms, users sought recommendations on common foods to prevent symptoms, manage flares, or promote fistula or abscess healing. One person sought advice on a safe cake for their birthday while others sought advice on food as IBD treatment. Reply posts shared advice for identifying food triggers, and in particular, patience with the process. Posts predominantly agreed that dietary plans need to be individually tailored and that while diet can mitigate symptoms, it is not sufficient to treat IBD.

### Domain 5: IBD’s Negative Impact on Mental Health (12% of Posts)

#### Seeking Advice on Comorbid Anxiety and Depressed Mood

Individuals frequently reported anxiety and depression as symptom triggers, but symptoms also triggered more anxiety. Others described a general sense of difficulty navigating their mental health in the context of their IBD. Some people specifically sought advice on mental health treatment in the context of IBD.

#### Sharing Feelings of Defeat and Embarrassment

Individuals expressed exhaustion and defeat as a result of common setbacks when living with IBD, including flare symptoms, medication adverse effects, and challenges in navigating care (eg, scheduling colonoscopies or access to gastroenterologists). Individuals sought reassurance that they were not alone in feeling defeated. Other posts described exhaustion specific to the impact of IBD on self-confidence, particularly given the invisibility of the disease. For example, one person expressed judgment by others when they were late or missed work due to their illness. Another post described embarrassment in having to ask for work accommodations due to pain and fecal urgency. Posts also described self-consciousness over physical manifestations, such as hair loss, acne, or weight gain. In response, reply posts provided validation of feelings and promoted positive thinking.

## Discussion

### Principal Findings

Individuals with IBD used the Reddit platform as a multifunctional tool for seeking disease-specific knowledge, advice, empathy, shared experiences, and validation for their health-related concerns, which are not often fully addressed through established clinical care. These discussions covered a number of domains, including symptoms as a major burden of living with their disease, concerns with medications and treatment choices, health care challenges, dietary regimens and dietary guidance for handling symptoms and as treatment, and the impact of IBD on mental health. The Lorig chronic disease model defines self-management as incorporating medical, role, and emotional management. These findings align with this established framework, with social media discussion focusing on key self-management tasks of treatment, mental health, challenges to navigating care, and the role of diet and symptoms in patients living with IBD.

### Comparison With Prior Work

These study findings align with previously published data in IBD. In an analysis of IBD-related posts on Facebook (Meta) and other popular social media networks, the top 5 topics of interest were diet, lifestyle, complementary and alternative medicine, diagnostic test interpretation, and specialist referrals and reviews. Many users in their adjunct survey reported high satisfaction with IBD care, which suggests the supporting role of these platforms in providing education and support to patients [19]. Further, in an analysis of ulcerative colitis flare-related online posts on 8 public forums, individuals most frequently discussed their treatment experiences and related side effects, as well as flare symptoms [11]. However, our findings expand on prior studies with a more comprehensive assessment of patients’ unmet needs in patients living with IBD. Our findings highlight the purpose of symptom-related posts, including sharing experiences, seeking advice, and seeking validation and empathy for symptoms, as well as responses from peers. In our study, individuals sought advice from others on social media about the normalcy of both bowel and extraintestinal symptoms and advice for symptom management, including dietary guidance. The contribution of dietary triggers to IBD symptoms often drives patients' eating behaviors and self-imposed restrictions [20]. Normalization of symptoms is one means of addressing patients’ concerns and is often a technique used in cognitive restructuring treatments [21].

Similar to published studies, our study highlights medication-related concerns and the exchange of medication advice within the IBD social media community. In our study, individuals sought advice from others on medication choices and related concerns. These concerns included potential medication adverse effects, treatment failure and uncertain next steps, as well as financial challenges with accessing medications. Interestingly, poor response to treatment and fear of adverse effects have been identified as key contributors to IBD-specific medication nonadherence using qualitative interview data [22]. There is also curiosity about nonstandard regimens and nonpharmacological alternatives to IBD treatment, which are common topics of discussion for patients with IBD [23]. The peer support function of social media also comes through in the reassurance provided by others to support patients in times of crisis, the sharing of successes with treatment response, and the emphasis on medication compliance and providing caution regarding false information around treatment de-escalation.

National survey data have shown that patients with IBD continue to experience barriers to accessing and affording high-quality IBD care, including access to health care professionals and medications [24]. Our analysis confirms that navigating health care is a major concern for patients with IBD. Individuals with IBD described difficulties establishing care with IBD-specialized gastroenterologists and communicating symptoms and problems with their medical team. Further, individuals’ perceived lack of empathy and willingness to adequately address concerns by the medical team led to hesitancy with seeking health care when needed. Insurance issues and variable health care coverage were also identified as a major challenge for patients with IBD. Therefore, medication affordability was a major source of frustration and anxiety for patients.

Stress and anxiety have also been identified as frequent IBD-related topics of discussion on social media (e.g., 38% of posts in some forums), and in particular, how these factors contribute to flare symptoms [11]. It is known that patients with IBD are at higher risk for anxiety and depression than patients without a diagnosis of IBD. The bidirectional relationship between perceived stress and IBD symptoms is well described. In a population-based registry study, higher perceived stress predicted higher symptom burden in the subsequent 3 months, and higher symptom burden also predicted increases in subsequent perceived stress [25]. Psychosocial interventions, particularly those grounded in cognitive behavioral therapy, have demonstrated efficacy in improving depression, quality of life, and disease activity in patients with IBD [26]. However, these interventions are difficult to disseminate widely and have not been adapted to the broader needs of patients with IBD.

Many social media users are not using Reddit as a primary source of medical advice, as discussions frequently referenced prior engagement with clinical care (eg, gastroenterologist and emergency services), and rather expressed a desire for seeking clarification, advice, shared experience, or support to complement these clinical interactions. This suggests that social media could serve as a supplemental tool for self-management of IBD. However, gastroenterologists should caution users about the reliability and validity of medical advice shared on these platforms. Overall, study findings identify several IBD-related topic areas for which patients seek support on social media, including symptoms, treatment, diet, mental health, and care navigation. While it may not be feasible to address all these concerns in the clinical context, these findings can inform targets for interventions to support patients in mastering the skills required for self-management. Systematic review data from self-management interventions have shown that building IBD-specific knowledge, promoting self-efficacy, and providing access to tools for monitoring medications and symptoms can improve IBD health. However, these study findings can inform further tailoring of these programs to specifically include content focused on these areas of need for patients with IBD [4].

### Strengths and Limitations

The major strengths of this study include the ability to capture experiences from a global sample given the reach of Reddit’s platform. In addition, the anonymity of the platform facilitates openness that may limit information gathered from interviews. Further, given the diversity of information provided from both original posts and reply posts, we were able to gather a breadth of qualitative data including the interaction between individuals in the community. However, this study also has several limitations. Although some users self-identified as having Crohn disease or ulcerative colitis, we were unable to identify IBD-specific demographic information or IBD type from posts or confirm that all participating users carry a true diagnosis of IBD, given the anonymity of Reddit data. Future studies should examine variation in experiences based on IBD type to inform the need for further tailoring of potential interventions. Given our qualitative analysis approach, we also did not capture every unique experience, rather major concepts. Finally, most Reddit platform users are under the age of 50 years, and individuals who post on social media may potentially have more symptoms, greater social needs, higher digital literacy, or carry less stigma to share their personal experience. Similarly, individuals who do not engage with Reddit or other online platforms may have different experiences from the Reddit users. Thus, study findings may not be generalizable to all patients with IBD [27].

### Conclusions

In conclusion, individuals with IBD face a diversity of experiences, challenges, and successes while managing their disease. The Reddit social media platform provides a valuable community for people to share their experiences, seek advice, gain IBD-specific knowledge, and receive empathy and reassurance. While social media platforms have provided an avenue for patients with IBD to gain knowledge, empathy, and support, the common use of social media for these needs suggests there is a critical gap in delivery of knowledge, empathy, and support for patients through established clinical practice. In addition, although social media platforms can play an important role in the exchange of information and advice related to living with IBD, there are several barriers to their use as an effective tool for supporting patients living with IBD. Prior studies have identified patient privacy, confidentiality, and lack of trust in posted information as major concerns related to social media use for health information among patients with IBD [28]. Therefore, there is a critical need for data-driven interventions to support patients in self-management and on-demand care that may be needed outside of a routinely scheduled office visit. These study findings can help support future efforts to develop these programs and inform prioritization of intervention targets, including management of symptoms, diet, medications, navigating health care, and mental health support. Further, these study findings identify areas for advocacy for the larger IBD community on a policy level, especially regarding insurance driving medication accessibility.

## Supplementary material

10.2196/75137 Multimedia Appendix 1 Checklist for Planning for and Assessing Rigor in Rapid Qualitative Analysis.

## Abbreviations

IBD: Inflammatory bowel disease
PARRQA: Planning for and Assessing Rigor in Rapid Qualitative Analysis
