# Supplementary material for: Reddit as a Social Media Self-Management Tool for Inflammatory Bowel Disease: Qualitative Analysis
Source: J Med Internet Res. 2025 Aug 1;27:e75137. doi: 10.2196/75137 (PMC12316438; doi:10.2196/75137)
Supplement: Multimedia Appendix 1 [file jmir-v27-e75137-s001.docx]

| **Table 1. Checklist for Planning for and Assessing Rigor in Rapid Qualitative Analysis (PARRQA)** | | |
| --- | --- | --- |
| **Item** | **Page Number** | **Guidelines to support rigor and validity** |
| **Rigorous Design** | | |
| 1 | Page 3-4 | Articulate research question and purpose |
| 2 | Page 4 | Describe the rationale for using rapid qualitative methods |
| 3 | Page 4 | Define what is meant by "rapid qualitative analysis" |
| 4 | Page 4 | Consider whether theory, model, or framework will be used to inform the study, and why/how |
| 5 | Page 4 | Define the intended timeframe of data collection, analysis, deliverables |
| 6 | Page 4 | Plan for appropriate staffing |
| 7 | Page 4 | Explain purpose and timeline of study, communication plan, roles |
| **Data Collection** | | |
| 8 | N/A | Develop and refine data collection tools to address specified research questions |
| 9 | N/A | Pilot data collection instruments to ensure they are clear, feasible, and appropriately targeted |
| 10 | N/A | Develop a plan for review throughout the data collection process |
| **RQA: Summary Template Development** | | |
| 11 | Page 4 | Develop and pilot test a user-friendly summary template |
| 12 | Page 4 | Ensure there are cross-references to raw data to support continuous comparison and validation |
| 13 | Page 4 | Develop summaries that are accurate and concise, but detailed enough to meet project aims |
| 14 | Page 4 | Identify training and calibration processes to ensure consistency and accuracy in summaries |
| **RQA: Matrix Analysis** | | |
| 15 | Page 4 | Plan qualitative matrix structure to reflect project aims/questions |
| 16 | Page 4 | Describe the use of software for matrix analysis (e.g., excel/word) |
| 17 | Page 4-5 | Develop a plan for review throughout matrix analysis |
| **Rapid Qualitative Data Synthesis** | | |
| 18 | Page 4-5 | Conduct synthesis that is rigorous and responsive to research priorities |
